# Novel *TBC1D8B* Variant in a 6-Month-Old Boy With Steroid-Sensitive Nephrotic Syndrome: A Case Report

**DOI:** 10.3389/fped.2021.732512

**Published:** 2021-11-11

**Authors:** Ling Hou, Lu Yin, Yubin Wu, Chengguang Zhao, Yue Du

**Affiliations:** Department of Pediatrics, Shengjing Hospital of China Medical University, Shenyang, China

**Keywords:** nephrotic syndrome, *TBC1D8B*, podocyte slit diaphragm protein, steroid-sensitive nephrotic syndrome, steroid-resistant nephrotic syndrome

## Abstract

A structural abnormality or dysfunction of podocytes is the major cause of nephrotic syndrome (NS). The TBC1D8B protein interacts with nephrin, a podocyte slit diaphragm protein, regulates vesicle transport, and functions in the pathogenesis of NS. We report a novel potentially pathogenic variant in the *TBC1D8B* gene in a 6-month-old boy with NS. A 6-month-old boy was admitted to the hospital because of edema and fever. Our systematic examination led to a diagnosis of NS. Because of the early age of onset, we performed trio whole-exome sequencing of him and his parents. The results showed a new potentially pathogenic variant in the *TBC1D8B* gene on the X chromosome, c.2717A>G (p.His906Arg). After routine glucocorticoid therapy, his urine protein turned negative, indicating steroid-sensitive NS. The new *TBC1D8B* variant identified here, c.2717A>G (p.His906Arg), may be associated with early-onset NS in children. Although NS due to pathogenic variants in this gene is more commonly steroid-resistant, our patient had steroid-sensitive NS.

## Introduction

Most children diagnosed with nephrotic syndrome (NS), defined by nephrotic-range proteinuria, hypoalbuminemia, edema, and hyperlipidemia, have idiopathic NS (INS), although there are reports of congenital NS. The incidence of NS is about 1.15–16.9 per 1-million children, and there are notable ethnic and geographic variations (1, 2). Immune system disorders, alterations in systemic circulation factors (a circulating glomerular permeability factor), and hereditary podocyte structural abnormalities may be responsible for the pathogenesis (3).

Podocytes, the glomerular basement membrane (GBM), and fenestrated endothelial cells normally form the glomerular filtration barrier. Podocytes are highly differentiated epithelial cells whose processes are interlaced and combined to form a slit diaphragm (SD). The maintenance of podocyte structure and function depends on interactions between the protein molecules that fix the podocytes on the GBM and maintain their unique structure, and interactions with glomerular endothelial cells (4). Therefore, damage of the podocytes affects the entire glomerular filtration barrier and leads to proteinuria.

TBC1D8B is a member of the TBC domain (tre2/Bub2/CDC16) protein family, whose gene is located at Xq22.3. A study in early 2019 first reported 2 cases with hemizygous pathogenic variants in the *TBC1D8B* gene who had steroid-resistant NS (SRNS) (5). A subsequent 2019 study reported five families with NS, four with SRNS, and one with steroid-sensitive NS (SSNS), that were caused by pathogenic variants in *TCB1DB8* (6). TBC1D8B is a Rab-GTPase activating protein that functions in endocytosis and circulation pathways. *TBC1D8B* affects the transport of the SD protein nephrin *via* Rab11-dependent vesicle transport and has a role in the pathogenesis of NS (5, 6).

In this study, we report a new hemizygous potentially pathogenic variant, c.2717A > G (p.His906Arg), in exon 17 of the *TBC1D8B* gene in a 6-month-old boy with NS.

## Case Presentation

### History of Illness, Examination, and Diagnosis

In December 2019, a 6-month-old boy was admitted to our hospital due to edema for 1 week and fever for 1 day. The child was the first child of a non-consanguineous marriage and with full term of 39W^+2^, was delivered by cesarean section, had a birth weight of 3,900 g, and had no remarkable details regarding medical or personal history. At admission, there was prominent edema on the whole body and pitting edema on the anterior tibial area. We diagnosed the patient with NS based on nephrotic-range proteinuria (263 mg/kg/day), obvious hypoalbuminemia (12.2 g/L). There were also several other abnormal laboratory results (Table 1). In particular, the coagulation function was altered with decreased antithrombins (AT) and slightly increased level of D-dimer and activated partial thromboplastin time (APTT). However, the prothrombin time (PT), fibrinogen degradation products (FDP), and fibrinogen (Fib) were in normal ranges (Table 1).

**Table 1:** Laboratory results of the child at admission.

**Variable**	**Value**	**Reference**	**Variable**	**Value**	**Reference**
***Peripheral blood cells***			Fib, g/L	3.8	2~4	
WBC, 10^9^/L	5.25	3.5~9.5	FDP, mg/L	3.4	0~5
RBC, 10^12^/L	3.6 ↓	4~4.5	D-dimer, μg/L	640 ↑	0~252
Hb, g/L	110 ↓	120~140	AT, %	16 ↓	83~128
Hct, %	28.3 ↓	37~47	***Serological tests***		
Plt, 10^9^/L	612 ↑	135~350	ANA	Negative	Negative
***Blood chemistry***			ANCA	Negative	Negative
TP, g/L	50.0 ↓	60~83	IgG, g/L	0.94 ↓	4.81~12.2
Alb, g/L	12.2 ↓	35~53	IgA, g/L	0.454	0.42~1.58
AST, U/L	24	5~34	IgM, g/L	1.49	0.41~1.65
ALT, U/L	10	0~40	C3, g/L	0.947	0.74~1.4
LDH, U/L	227	103~227	C4, g/L	0.785 ↑	0.12~0.36
BUN, mmol/L	5.91	2.5~7.2	ASO, IU/mL	<25	0~200
Cr, μmol/L	34.1	45~84	FT3, pmol/L	<1.54 ↓	2.63~5.71
Na, mmol/L	119 ↓	135~145	FT4, pmol/L	<5.15 ↓	9.01~19.05
K, mmol/L	5.0	3.5~5.5	TSH, μIU/mL	9.7711 ↑	0.30~4.80
Cl, mmol/L	106	96~108	Anti-Tg, IU/mL	0.251	0~4.11
TG, mmol/L	7.66 ↑	0.4~1.69	Anti-TPO, IU/mL	0.00	0~5.61
Chol, mmol/L	9.75 ↑	3.36~5.69	***Urinalysis***		
CRP, mg/L	1.03	0~8	Gravity	1.020	1.003~1.030
HCY, μmol/L	12.17	0~15	pH	6.50	4.5~8.0
CER, g/L	0.228 ↓	0.31~0.55	Protein	3+ ↑	Negative
PT, s	11.9	9.4~12.5	RBC, /HP	312 ↑	0~3
APTT, s	55 ↑	21~37	Proteinuria, g/day	6.24 ↑	0~0.15

The ultrasound showed enlarged kidneys (the left and right kidney were 8.5 × 4.9 × 4.0 cm and 8.6 × 4.9 × 3.7 cm, respectively) with hyperechogenicity of renal parenchyma, blurred cortical and medullary boundaries. The depth of pelvic and abdominal cavity effusion was 3.4 cm. A chest computerized tomography (CT) showed multiple bilateral pulmonary foci, partial pulmonary consolidation, bilateral pleural effusion, and poorly inflated adjacent pulmonary alveoli. His heart function was normal and the nervous system had no abnormalities. The routine urine test results of both parents were normal.

### General Symptomatic Treatment

We prescribed ceftriaxone for anti-infective treatment and other adjuvant therapies, such as infusion of plasma and albumin, to increase the colloidal osmotic pressure and manage the diuresis. He received the administration of intravenous enoxaparin sodium 0.06 ml/day (50 AxaIU/kg/day) for anticoagulant therapy at admission because of severe hypoalbuminemia and complicated fluid management. We also administered intravenous immunoglobulin intermittently to provide immune support. The levels of free triiodothyronine (FT3) and free thyroxine (FT4) were significantly reduced, and the level of thyroid-stimulating hormone (TSH) was significantly increased, but there were no evident abnormalities in ultrasound. The diagnosis of hypothyroidism was considered, and we administered oral levothyroxine sodium (12.5 μg/day).

### Immunosuppressive Therapy

The child developed an intestinal infection caused by norovirus after hospitalization. Due to his young age and this infection, after excluding tuberculosis, we administered intravenous methylprednisolone (1.6 mg/kg/day) on the 4th day in the hospital. After 25 days of hospitalization, the child's general condition improved significantly, the edema subsided, the 24-h urine protein had decreased significantly (0.43 g/day), and the plasma albumin increased to 20.5 g/L. Analysis of coagulation showed that AT, D-dimer, APTT, PT, FDP, and Fib returned to their normal ranges. The child was discharged and continued to take adequate oral prednisone (2 mg/kg/day) for 2 weeks with a gradually reducing dosage (2 and 0.5 mg/kg/day alternatively in the morning) for 1 month. After that 2 mg/kg/day on an alternate day for 2 weeks, then reduce 2.5 mg every 2 weeks.

### Whole-Exome and Mitochondrial Genetic Sequencing

**Figure 1:**
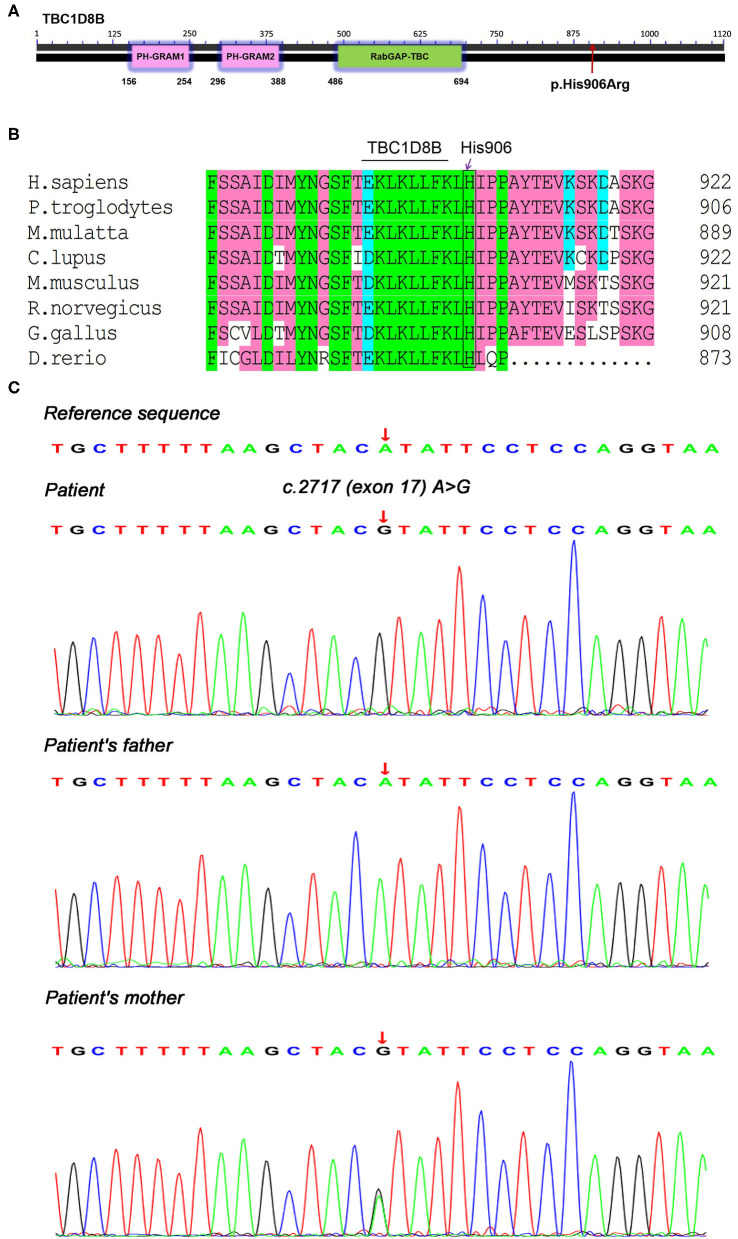
Genetic testing and conservation of the *TBC1D8B* sequence in different species. **(A)** TBC1D8B protein structure and location of the child's p.His906Arg mutation, **(B)** amino acid sequence conservation in different species, and **(C)** trio whole-exome sequencing results, showing a hemizygous mutation, c.2717A>G (p.His906Arg), in the *TBC1D8B* gene of the child and the mother.

Because of the young age at disease onset, we considered the child might have had congenital NS. Therefore, we performed trio whole-exome and mitochondrial genetic sequencing on the 5th day in the hospital. Briefly, 3 mL of peripheral blood was collected from the patient and his parents, and genomic DNA was extracted using a blood genome extraction kit (Tiangen). Capture sequencing was performed using the human total exome capture probe of JOY Orient. The capture probe covered the coding region of 19,396 genes in the human genome, and the capture target interval was 39 MB. A total of 429,826+ capture probes were designed and synthesized, and the capture probe covered 51 M. Sequencing was performed using the Novase Q6000 instrument. After obtaining the original sequenced reads, fine filtering of raw reads was required. Clean reads obtained in this way were compared with HG19/GRCH37 (reference genome). Software (GATK) was used for detection and analysis of single nucleotide polymorphisms and InDels. The coverage of the target area was 99.90%, and the average depth was 109-fold. The results indicated a novel hemizygous potentially pathogenic variant, c.2717A>G (p.His906Arg), in exon 17 of the *TBC1D8B* gene. This potentially pathogenic variant was derived from the mother (who had no history of symptoms and no proteinuria) and the father's gene was normal (Figure 1). The amino acid sequences for this site (His906) showed that it was identical in *Homo sapiens, Pan troglodytes, Macaca mulatta, Canis lupus familiaris, Mus musculus, Rattus norvegicus, Gallus gallus*, and *Danio rerio* (Figure 1). The minor allele frequency (MAF) of this site in the gnomAD database was 0.0005 (East Asia). Protein structure and function prediction software indicated the variant was probably damaging (Polyphen) or deleterious (SIFT).

### Follow-Up

The child was followed up for 1 year (Table 2) and treated with oral prednisone, vitamin D, and calcium supplementation. During this follow-up period, the boy experienced 2 relapses of NS when alternate-day morning prednisone was reduced to 17.5 mg (March 2020), and the second recurrence was accompanied by a fever (June 2020) which indicated steroid-dependent NS. Adequate oral prednisone (20 mg/day) was given to achieve remission, followed by dose reduction. As of November 2020, the dose of alternate-day prednisone was 5 mg. The urine test results were normal (protein negative, 2.12 RBCs/HPF), and the 24-h urine protein was 0.12 g/day. The child's serum albumin returned to normal (42.6 g/L), as did his renal function (blood urea nitrogen, 2.87 mmol/L; serum creatinine: 17.5 μmol/L). And the patient didn't show any signs of steroid toxicity. We reduced the oral levothyroxine sodium dose to 6.25 μg/day, and measurements of thyroid function were in the normal ranges (FT3 5.71 pmol/L; FT4, 12.4 pmol/L; TSH, 0.7323 μIU/mL).

**Table 2:** Changes of renal function parameters and other clinical parameters during the course of disease.

**Variable**	**Admission**	**Discharge**	**Follow-up**		
			**3 months**	**6 months**	**1 year**
Weight	12.0 kg	9.0 kg	9.6 kg	10.5 kg	12.6 kg
Height	64 cm	64 cm	68 cm	74 cm	80 cm
Blood pressure	90/49 mmHg	97/64 mmHg	96/55 mmHg	92/63 mmHg	92/55 mmHg
Urinary protein	3+	±	–	–	–
Urinary RBC, /HP	312	2.16	2.01	2.07	2.12
Proteinuria, g/day	1.58	0.43	0.12	0.13	0.12
Alb, g/L	12.2	20.5	36.8	38.6	42.6
BUN, mmol/L	5.91	2.42	2.0	3.33	2.87
Cr, μmol/L	34.1	14.2	18.2	21.6	17.5
FT3, pmol/L	<1.54	3.23	5.74	5.39	5.71
FT4, pmol/L	<5.15	12.37	12.33	12.51	12.4
TSH, μIU/mL	9.7711	2.1181	0.8240	0.7787	0.7323
Prednisone	16 mg/day; intravenous methylprednisolone	20 mg/day	17.5 mg alternate days	15 mg alternate days	5 mg alternate days
Levothyroxine sodium	12.5	12.5	10	6.25	6.25

## Discussion

*TBC1D8B*, like other TBC proteins, has two conserved amino acid residues at the active center, which binds to a specific Rab protein and stimulates its GTPase activity. This protein is thus considered a Rab-GTPase activating protein (7). In addition to its Rab-GAP domain, TBC1D8B also contains two GRAM domains that bind to lipid rafts, a key element of SD signaling in podocytes (8).

A 2019 study first reported that pathogenic variants in the *TBC1D8B* gene were associated with SRNS. One boy (from an Ecuadorian family) had the p.Gln246His variant and developed NS within 3 months after birth. The other boy (from a European family) had the p.Phe291Ser variant and developed NS at 2 years. Both of these variants were in the GRAM domain (5). Alterations in the structure of *TBC1D8B* could lead to structural changes and defects in podocyte migration. Studies of zebrafish reported that knockdown or knockout of the *TBC1D8B* homologous gene induced proteinuria and that this was rescued by injecting normal human *TBC1D8B* mRNA, but not mutant mRNA. There is also evidence of an interaction between TBC1D8B and the essential protein Rab11b in cell vesicle circulation (5).

Kampf et al. subsequently reported hemizygous missense pathogenic variants in the *TBC1D8B* gene in five families with NS. These families were from Spain, Turkey, Germany, and Pakistan, and four of the patients had SRNS. The boys developed NS, but the girls only had mild proteinuria. Only one pathogenic variant was located in the GRAM domain, and the other pathogenic variants were not in the GRAM domain or the Rab-GAP domain (6). In particular, a patient with a p.Arg64Cys variant, a highly conserved region, is classified as having SSNS with an age of 7 years at diagnosis (6). Kampf et al. found that silencing *TBC1D8B* in HEK293T cells increased basal autophagy and extracellular secretion. TBC1D8B also interacts with the SD protein nephrin, and regulates vesicle trafficking. Over-expression of mutated mouse *Tbc1d8b* reduced the protein's affinity to endogenous Rab11 and nephrin, and knockout of the *Tbc1d8b* gene in Drosophila impaired the function of podocyte-like kidney cells (6).

Our child had early-onset NS and a new hemizygous potentially pathogenic variant at exon 17 in the *TBC1D8B* gene. Amino acid sequence analysis indicated this locus was highly conserved, and maybe the potentially genetic basis for this child's condition. Our literature review found that most children with hemizygous pathogenic variants in the *TBC1D8B* gene had SRNS, although one previous child with a p.Arg64Cys variant had SSNS. There were also variations in the age of onset, with one patient experiencing onset at the age of 7 years. The *TBC1D8B* gene has two transcripts (*TBC1D8B-a* and *TBC1D8B-b*). The *TBC1D8B-b* transcript is unique to humans and codes for a protein of 632 AA; the *TBC1D8B-a* transcript codes for a protein of 1120 AA. This may lead to the hypothesis that a variant in a region unique to the *TBC1D8B-a* transcript (coding for AA 633–1120) leads to SSNS, as in our patient, and that a variant in a region common to both transcripts leads to SRNS, as in previous patients (5, 6). We must reject this hypothesis because there is also evidence that the p.Arg64Cys variant (both transcripts) led to SSNS and the p.Thr780Ser variant (*TBC1D8B-a* transcript alone) led to SRNS (6). Therefore, variants of different transcripts seem to have little effect on the clinical phenotype. And we should keep in mind that the potentially pathogenic variant we found might not be pathogenic because of the lack of basic experiments to support our idea. It will be helpful to observe this variant in different populations worldwide.

Our patient had obviously decreased levels of FT3 and FT4, but a thyroid ultrasound indicated no apparent abnormalities at admission. Thyroid hypofunction may be associated with a low level of serum albumin caused by NS. Considering the early age of onset and the unclear effect of steroid treatment, we administered a low dose of oral levothyroxine sodium (12.5 μg) to maintain normal growth and development. As his condition improved, we gradually reduced the dose of levothyroxine sodium to 6.25 μg, and plan to discontinue this treatment in the near future.

## Conclusion

This study is the first report of a hemizygous potentially pathogenic variant, c.2717A>G (p.His906Arg), in the *TBC1D8B* gene in a child with SSNS from a Chinese family. This variant may be the potentially genetic basis of the child's pathogenesis.

## Data Availability Statement

The raw data supporting the conclusions of this article will be made available by the authors, without undue reservation.

## Ethics Statement

Ethical review and approval was not required for the study on human participants in accordance with the local legislation and institutional requirements. Written informed consent to participate in this study was provided by the participants' legal guardian/next of kin. Written informed consent was obtained from the minor(s)' legal guardian/next of kin for the publication of any potentially identifiable images or data included in this article.

## Author Contributions

LH reviewed the literature and contributed to manuscript writing. CZ, YW, and LY contributed to the acquisition and analysis of the clinical data. YD was responsible for revision of the manuscript. All authors read and approved the final manuscript.

## Funding

This work was supported by grants from the Natural Science Foundation of Liaoning Province, China (2020-BS-116).

## Conflict of Interest

The authors declare that the research was conducted in the absence of any commercial or financial relationships that could be construed as a potential conflict of interest.

## Publisher's Note

All claims expressed in this article are solely those of the authors and do not necessarily represent those of their affiliated organizations, or those of the publisher, the editors and the reviewers. Any product that may be evaluated in this article, or claim that may be made by its manufacturer, is not guaranteed or endorsed by the publisher.

## Abbreviations

NS: nephrotic syndrome
INS: idiopathic NS
GBM: glomerular basement membrane
SD: slit diaphragm
SRNS: steroid-resistant NS
SSNS: steroid-sensitive NS
CT: computerized tomography
TSH: thyroid-stimulating hormone.
